# FGF21 Counteracts Alcohol Intoxication by Activating the Noradrenergic Nervous System

**DOI:** 10.1016/j.cmet.2023.02.005

**Published:** 2023-03-07

**Authors:** Mihwa Choi, Marc Schneeberger, Wei Fan, Abhijit Bugde, Laurent Gautron, Kevin Vale, Robert E. Hammer, Yuan Zhang, Jeffrey M. Friedman, David J. Mangelsdorf, Steven A. Kliewer

**Affiliations:** 1 Department of Pharmacology, UT Southwestern Medical Center, Dallas, TX 75390, USA.; 2 Laboratory of Molecular Genetics, Howard Hughes Medical Institute, The Rockefeller University, New York, NY 10065, USA.; 3 Live Cell Imaging Core Facility, UT Southwestern Medical Center, Dallas, TX 75390, USA.; 4 Division of Hypothalamic Research, Department of Internal Medicine, UT Southwestern Medical Center, Dallas, TX 75390, USA.; 5 Department of Biochemistry, UT Southwestern Medical Center, Dallas, TX 75390, USA.; 6 Howard Hughes Medical Institute, UT Southwestern Medical Center, Dallas, TX 75390, USA.; 7 Department of Molecular Biology, UT Southwestern Medical Center, Dallas, TX 75390, USA.; 8 Lead contact; 9 Present address: Department of Cellular and Molecular Physiology, Yale University, New Haven, CT 06510, USA.

**Keywords:** FGF21, hormone, alcohol, inebriation, anti-intoxicant, amethystic, liver, noradrenergic, locus coeruleus

## Abstract

Animals that consume fermenting fruit and nectar are at risk of exposure to ethanol and the detrimental effects of inebriation. In this report, we show that the hormone FGF21, which is strongly induced by ethanol in murine and human liver, stimulates arousal from intoxication without changing ethanol catabolism. Mice lacking FGF21 take longer than wild-type littermates to recover their righting reflex and balance following ethanol exposure. Conversely, pharmacologic FGF21 administration reduces the time needed for mice to recover from ethanol-induced unconsciousness and ataxia. FGF21 did not counteract sedation caused by ketamine, diazepam or pentobarbital, indicating specificity for ethanol. FGF21 mediates its anti-intoxicant effects by directly activating noradrenergic neurons in the locus coeruleus region, which regulates arousal and alertness. These results suggest that this FGF21 liver-brain pathway evolved to protect against ethanol-induced intoxication and that it might be targeted pharmaceutically for treating acute alcohol poisoning.

## eTOC

Choi et al. show that the alcohol-induced hormone FGF21 signals from liver to brain to counteract loss of consciousness and coordination caused by ethanol consumption. Pharmacologic FGF21 administration further accelerates recovery from inebriation. FGF21 exerts its sobering effect by activating the noradrenergic nervous system, which regulates arousal and alertness.

## Graphical Abstract

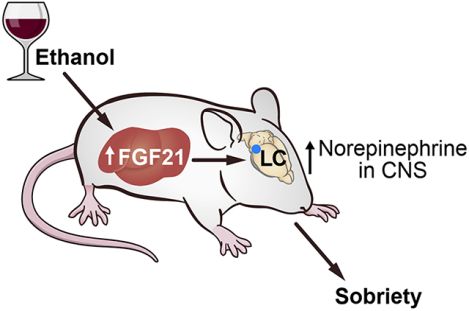

## INTRODUCTION

Simple sugars in ripening fruits and nectars are a rich source of calories for many animals. However, consumption of ethanol produced by the natural fermentation of these sugars can cause intoxication, thus impairing mobility and judgement^1^. Accordingly, animals that consume fructose and other simple sugars have evolved liver enzymes to catabolize ethanol. Comparative genetic analyses of alcohol dehydrogenases across species reveal that many strict herbivores and carnivores that are not exposed to ethanol appear to have lost the ability to catabolize it, underscoring the importance of ethanol as an evolutionary driver^2^.

FGF21 is a hormone that is induced in liver by a variety of metabolic stresses including starvation, protein deficiency, simple sugars and ethanol^3,4^. In humans, ethanol is by far the most potent inducer of FGF21 described to date^5–7^. Previous studies showed that FGF21 suppresses ethanol preference^8–10^, induces water drinking to prevent dehydration^7^, and protects against alcohol-induced liver injury^5,11,12^. Thus, FGF21 plays a broad role in defending against the harmful consequences of ethanol exposure.

FGF21 acts on a heteromeric cell surface receptor composed of a conventional FGF receptor tyrosine kinase (FGFR1c) in complex with the single-pass transmembrane protein, β-Klotho (KLB)^13^. FGF21 binds directly to both FGFR1c and KLB, with FGFR1c serving as the downstream signaling effector. Human genome-wide association studies have linked SNPs in and around both the *FGF21* and *KLB* genes to alcohol consumption^8,14,15^, further highlighting the important relationship between FGF21 and ethanol. In mice, FGF21 crosses the blood-brain-barrier through an unknown mechanism^16^ and acts on its receptor complex in the nervous system both to suppress ethanol preference and to induce water consumption^7,8^.

Norepinephrine (NE) is an abundant neuromodulator in the CNS. Most central NE is synthesized in the locus coeruleus (LC), a small nucleus in the pons of the brainstem. LC neurons project extensively throughout the brain to regulate diverse biological processes, including arousal and alertness^17,18^. In this report, we show that FGF21 directly activates noradrenergic neurons in the LC region. We further show that this FGF21-NE pathway accelerates recovery of righting reflex and balance following ethanol intoxication. Thus, FGF21 is an endogenous anti-intoxicant or “amethystic” agent (Alkana and Noble, 1979).

## RESULTS

### FGF21 deficiency exacerbates ethanol-induced intoxication

**Figure 1.**
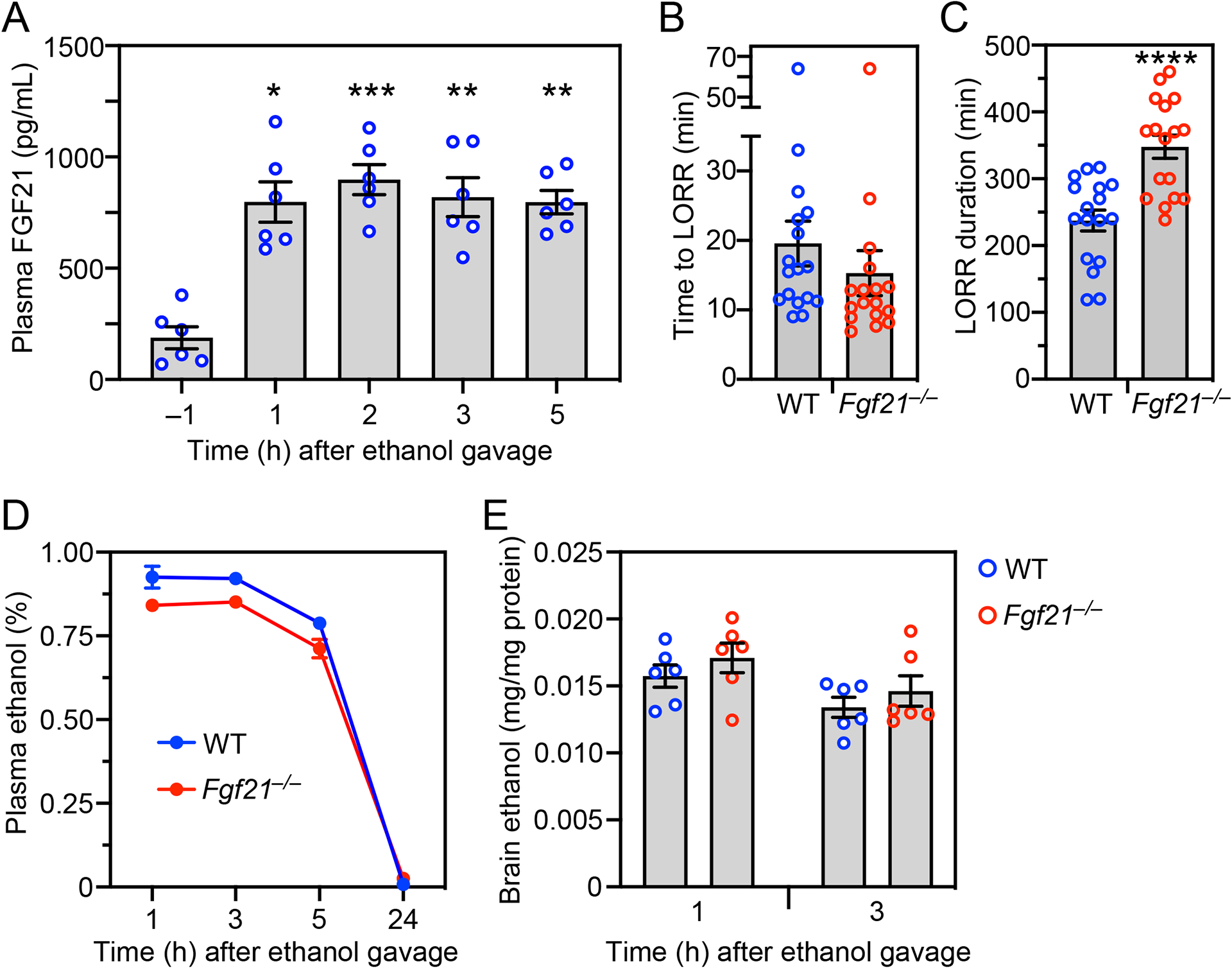
*Fgf21*^−/−^ mice have a prolonged righting reflex recovery time after a binge ethanol dose. (A) Plasma FGF21 concentrations were measured in wild-type (WT) mice either 1 hour before administering ethanol (5 g/kg, oral gavage) or at the indicated times after ethanol (n = 6 mice/group). (B-E) WT and *Fgf21*^−/−^ mice were administered ethanol (5 g/kg, oral gavage) and the following measurements made: time to loss of righting reflex (LORR) (B); LORR duration (C); plasma ethanol concentrations (D); brain ethanol concentrations (E) (n = 17 mice/group for (B) and (C) and 6 mice/group for (D) and (E)). All data represent the mean ± SEM. *, *P* < 0.05, **, *P* < 0.01, ***, *P* < 0.001 compared to −1 hour (A; one-way ANOVA) or WT (C; Student’s t-test).

We administered a single, binge dose of ethanol (5 g/kg by oral gavage) to wild-type (WT) and global *Fgf21*^*–/–*^ mice. Consistent with prior results, plasma FGF21 was induced by ethanol in WT mice, peaking at 2 hours (Figure 1A). We next examined the animals’ righting reflex, a standard marker of inebriation. While both WT and *Fgf21*^*–/–*^ mice lost their righting reflex 15–20 minutes after ethanol gavage (Figure 1B), *Fgf21*^*–/–*^ mice required ~1.5 hours longer to recover it than WT mice (Figure 1C). WT and *Fgf21*^*–/–*^ mice cleared ethanol from the plasma at the same rate (Figure 1D), and brain ethanol concentrations were similar between the genotypes (Figure 1E). Thus, FGF21 protects against ethanol-induced loss of righting reflex without affecting ethanol catabolism.

**Figure 2.**
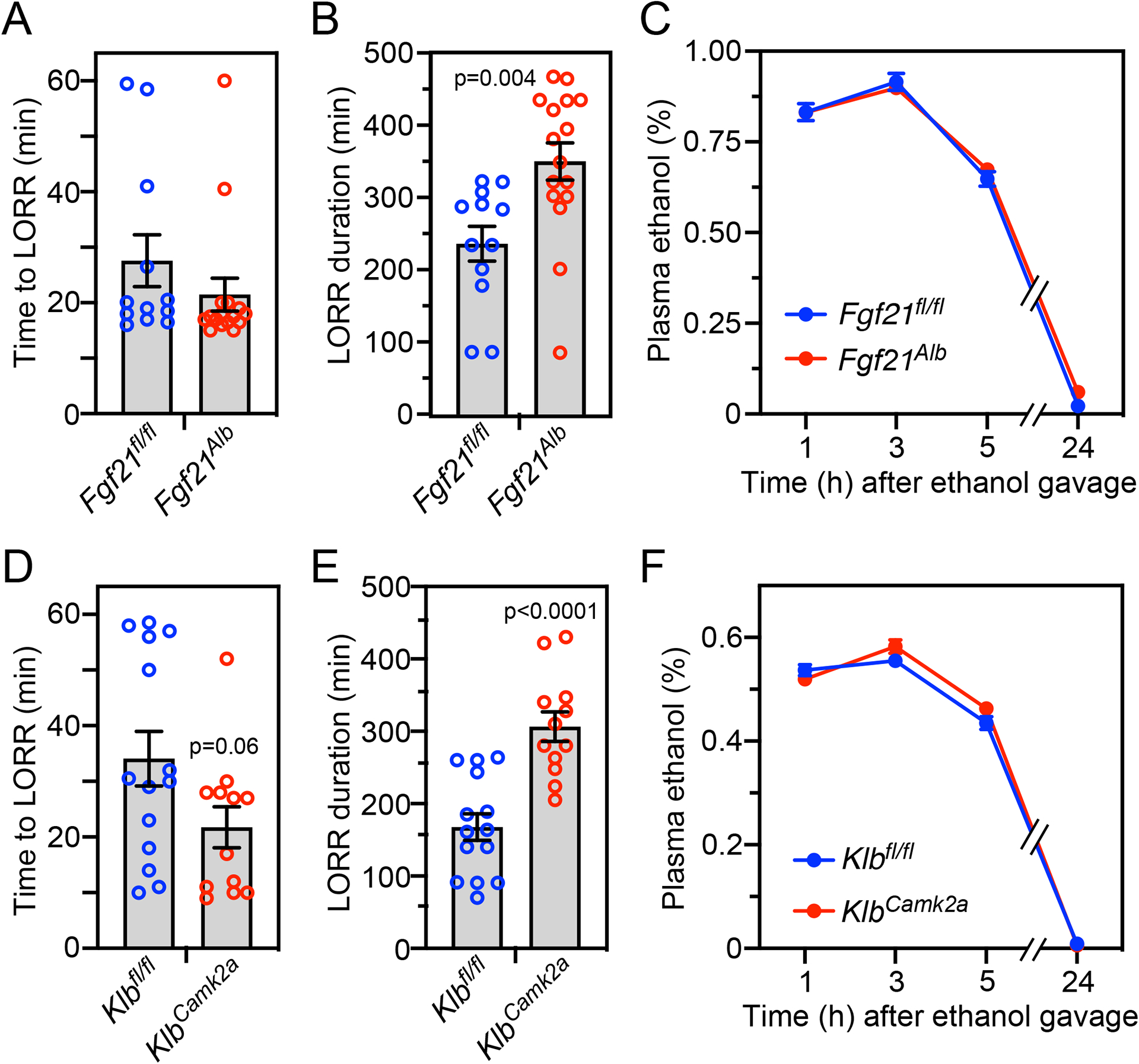
Hepatocyte-specific *Fgf21*^−/−^ and neuron-specific *Klb*^−/−^ mice have prolonged righting reflex recovery times after a binge ethanol dose. Control (*Fgf21*^*fl/fl*^) and hepatocyte-specific *Fgf21*^−/−^ (*Fgf21*^*Alb*^) mice (A-C) or control (*Klb*^*fl/fl*^) and neuron-specific *Klb*^−/−^ (*Klb*^*Camk2a*^) mice (D-F) were administered ethanol (5 g/kg, oral gavage) and the following measurements made: time to loss of righting reflex (LORR) (A, D); LORR duration (B, E); and plasma ethanol concentrations (C, F) (n = 12–16 mice/group for A, B, D, E and 11–12 mice/group for C and 10–11 mice/group for F). All data represent the mean ± SEM. *P* values in (B, D, E) by Student’s t-test.

We obtained similar results for both time to loss of righting reflex and its duration with hepatocyte-specific *Fgf21*-knockout (*Fgf21*^*Alb*^) mice (Figures 2A and 2B) and neuron-specific *Klb*-knockout (*Klb*^*Camk2a*^) mice (Figures 2D and 2E). There were no differences in plasma ethanol clearance between the knockout lines and control mice (Figures 2C and 2F). These results indicate that liver-derived FGF21 accelerates recovery from ethanol-induced loss of righting reflex by acting on its receptor in the nervous system.

### Pharmacologic FGF21 has anti-intoxicant activity

**Figure 3.**
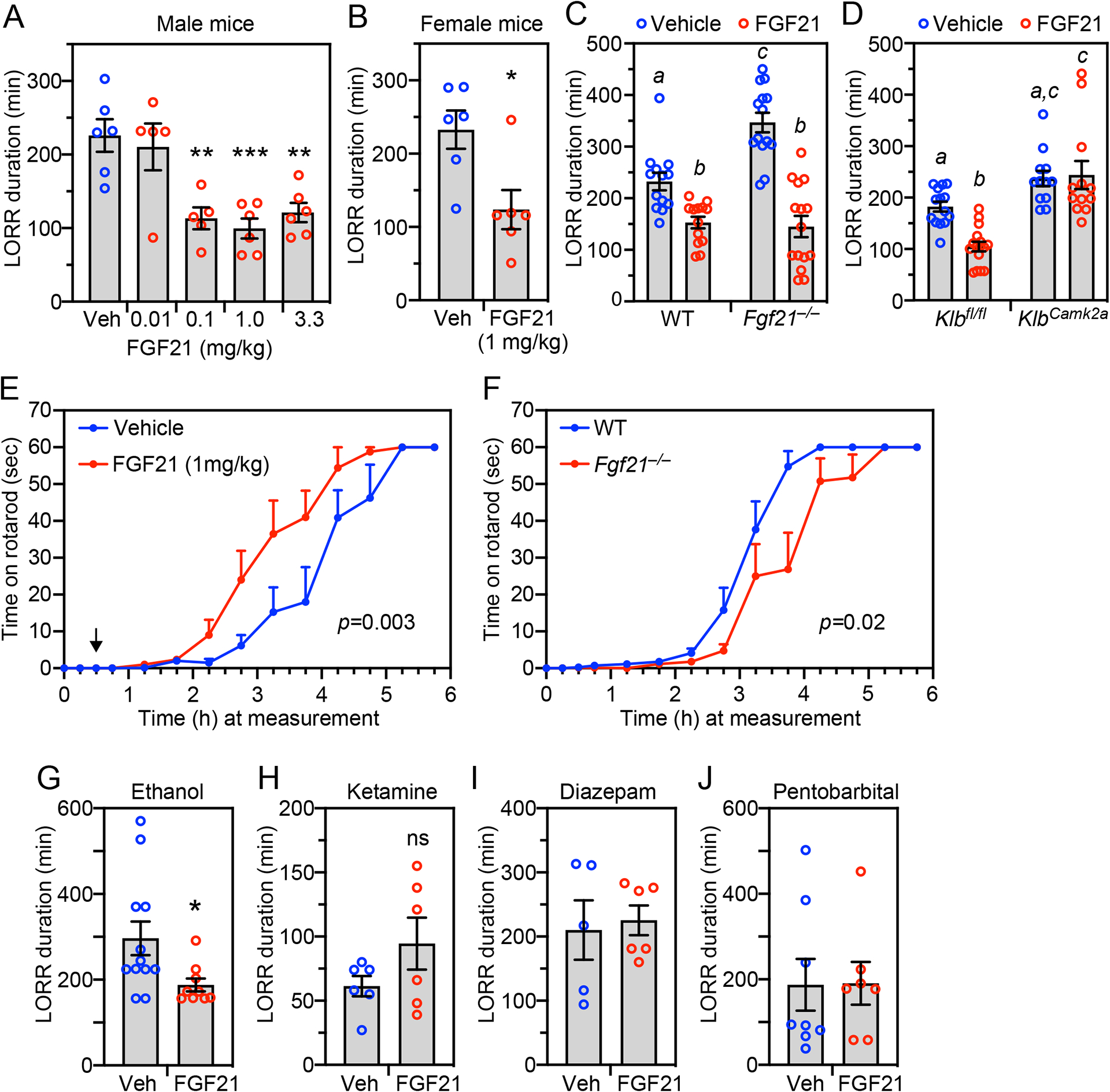
Pharmacologic FGF21 accelerates recovery from alcohol-induced loss of righting reflex and ataxia and is selective for ethanol. (A, B) Wild-type (WT) male (A) or female (B) mice were administered ethanol (5 g/kg, oral gavage) followed 1 hour later by i.p. injection of vehicle or FGF21 at the indicated doses. Loss of righting reflex (LORR) duration was measured after FGF21 or vehicle administration (n = 5–6 mice/group). Data represent the mean ± SEM. *, *P* < 0.05, **, *P* < 0.01, ***, *P* < 0.001 compared to vehicle by one-way ANOVA (A) or Student’s t-test (B). (C, D) WT and *Fgf21*^−/−^ mice (n = 13–15 mice/group) (C) or control (*Klb*^*fl/fl*^) and neuron-specific *Klb*^−/−^ (*Klb*^*Camk2a*^) mice (n = 12–16 mice/group) (D) were administered ethanol (5 g/kg, oral gavage) followed 1 hour later by i.p. injection of FGF21 (1 mg/kg) or vehicle. LORR duration was measured after FGF21 or vehicle administration. Data represent the mean ± SEM. Different lowercase letters indicate statistical significance (*P* < 0.05 by two-way ANOVA). (E) WT mice were administered ethanol (2 g/kg, i.p.) followed 30 minutes later by injection of FGF21 (1 mg/kg, i.p.; indicated by arrow) or vehicle. The time mice could remain on a spinning rotarod was measured, with 60 seconds the maximum (n = 8 mice/group). Data represent the mean ± SEM. *P* value by likelihood ratio test as described in Methods. (F)WT and *Fgf21*^−/−^ mice were administered ethanol (2 g/kg, i.p.) and the time they could remain on a spinning rotarod measured as in (E) (n = 8 mice/group). Data represent the mean ± SEM. *P* value by likelihood ratio test as described in Methods. (G-J) Wild-type mice were administered ethanol (4.3 g/kg) (G) (n = 9–12 mice/group), ketamine (200 mg/kg) (H) (n = 6 mice/group), diazepam (30 mg/kg) (I) (n = 5–6 mice/group) or pentobarbital (55 mg/kg) (J) (n = 7–8 mice/group) by i.p. injection. After 30 minutes, mice were i.p. injected with either FGF21 (1 mg/kg) or vehicle. Loss of righting reflex (LORR) duration was measured after FGF21 or vehicle administration. Data represent the mean ± SEM. *, *P* < 0.05 compared to vehicle-treated mice by Student’s t-test.

We next examined whether pharmacologic FGF21 treatment of WT mice stimulates righting reflex recovery following ethanol administration. WT mice were administered a binge ethanol dose (5 g/kg by oral gavage) followed one hour later, when the mice were already unconscious, by i.p. injection of recombinant FGF21. Remarkably, FGF21 administration reduced the time required for both male and female mice to recover their righting reflex by ~1.5 hours (Figures 3A and 3B), reflecting a roughly 50% decrease compared to vehicle-treated mice. This effect was dose dependent and maximally efficacious at 1 mg/kg FGF21 (Figure 3A), a standard pharmacologic dose that results in a maximum serum concentration of ~1500 ng/ml with a half-life of 1.3 hours^19^. Thus, circulating FGF21 concentrations are super-physiologic for the duration of the experiments.

We performed this same pharmacologic FGF21 rescue experiment in *Fgf21*^−*/*−^ and *Klb*^*Camk2a*^ mice. In *Fgf21*^−*/*−^ mice, FGF21 administration reduced the righting reflex recovery time to that seen in WT mice (Figure 3C). In contrast, FGF21 had no effect on righting reflex recovery time in *Klb*^*Camk2a*^ mice (Figure 3D), demonstrating that pharmacologic FGF21 exerts its amethystic effect via the nervous system.

We also investigated whether FGF21 inhibits ethanol-induced impairment of motor coordination. Pharmacologic FGF21 treatment reduced the time required for WT mice to recover their coordination on a rotarod following administration of a moderate dose of ethanol (2 g/kg, i.p.) (Figure 3E). Conversely, recovery time was significantly increased in *Fgf21*^−*/*−^ compared to WT mice (Figure 3F). Thus, pharmacologic FGF21 accelerates recovery from ethanol-induced intoxication as measured by both righting reflex and rotarod performance.

### FGF21’s anti-intoxicant activity is selective for ethanol

We next tested whether FGF21 counteracts other sedatives, including the glutamatergic receptor antagonist ketamine and the GABA receptor agonists diazepam and pentobarbital. Because these sedatives act more quickly and for a shorter duration than ethanol, we compressed the experimental timeline: mice were i.p. injected with either ethanol or each of the other sedatives followed by i.p. injection of FGF21 or vehicle 30 minutes later. FGF21 retained its ability to accelerate righting reflex recovery in ethanol-treated mice under these modified conditions (Figure 3G). In contrast, FGF21 administration did not stimulate recovery from ketamine, diazepam and pentobarbital sedation (Figures 3H–J). Thus, FGF21’s amethystic activity is selective for ethanol.

### FGF21 is a physiologic regulator of noradrenergic neurons

**Figure 4.**
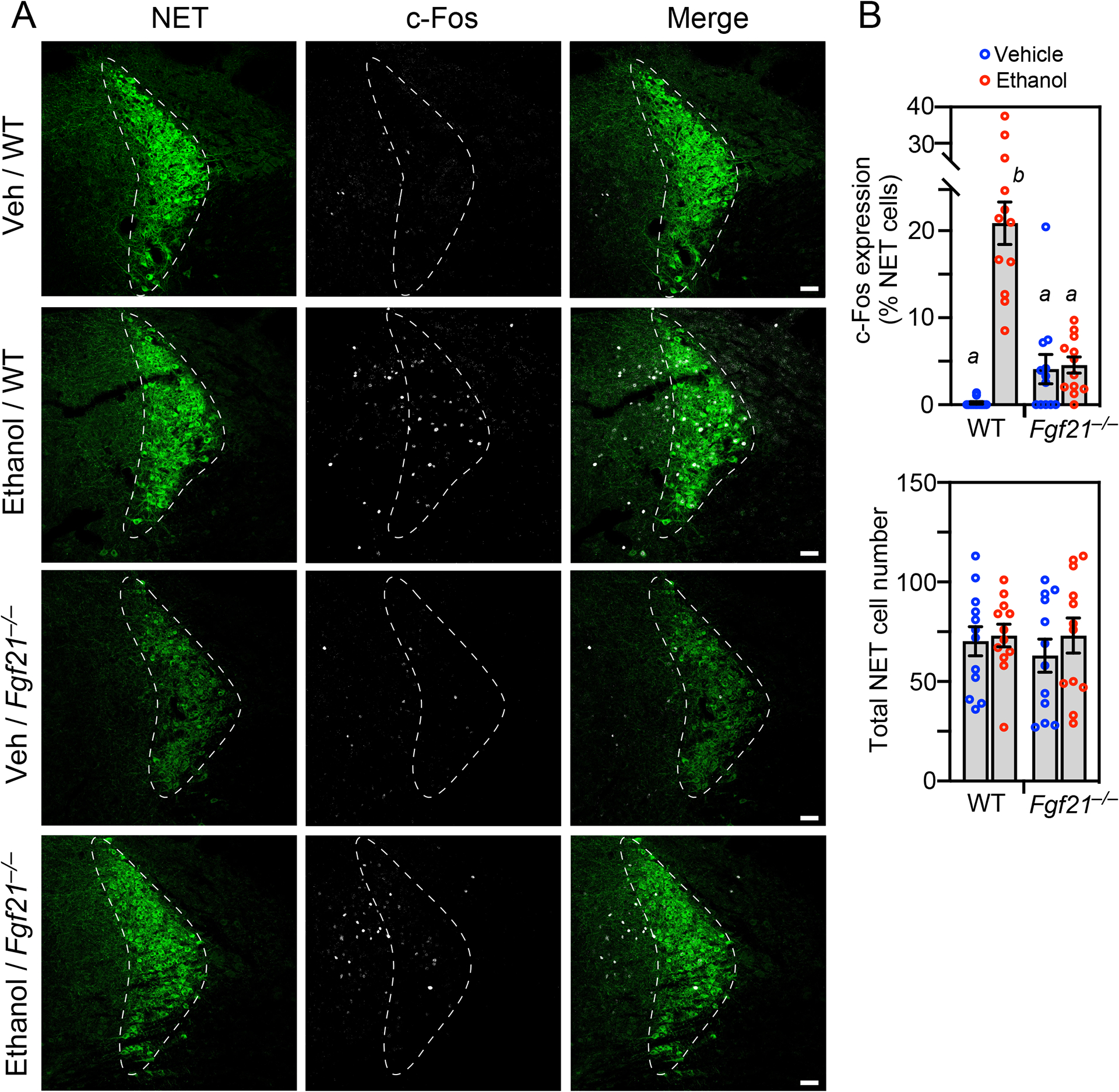
FGF21 is a physiologic regulator of noradrenergic neurons. (A) Representative confocal images of immunostaining for c-Fos (white) and norepinephrine transporter (NET; green) in locus coeruleus sections prepared from wild-type (WT) and *Fgf21*^−**/**−^ mice 2.5 hours after oral gavage with either water or ethanol (5 g/kg). Scale bar represents 50 μM. (B) Quantification of c-Fos/NET co-expression (upper panel) and total NET-positive cell number (lower panel) (n = 3 sections/mouse, 4 mice/group). Data represent the mean ± SEM. Different lowercase letters indicate statistical significance (*P* < 0.05) by two-way ANOVA.

Previous studies in mice and rats showed that ethanol administration activates neurons in the LC, the principal site of NE synthesis^20–22^. Moreover, dopamine β-hydroxylase (*Dbh*)-KO mice, which are unable to synthesize NE, have a prolonged righting reflex recovery time in response to ethanol without any change in ethanol catabolism^23^, similar to the responses we observed in *Fgf21*^−*/*−^ mice. These findings led us to investigate whether physiologic FGF21 is responsible for ethanol-induced activation of noradrenergic neurons in the LC. WT and *Fgf21*^−*/*−^ mice were administered ethanol (5 g/kg) by oral gavage and sacrificed 2.5 hours later. Immunostaining of LC sections was performed for c-Fos and the NE transporter (NET), which are markers of neuronal activity and noradrenergic neurons, respectively. As previously reported, ethanol induced c-Fos expression in NET^+^ LC neurons of WT mice (Figures 4A and 4B)^20–22^. Remarkably, this effect was completely absent in *Fgf21*^−*/*−^ mice (Figures 4A and 4B). The total number of NET^+^ cells was equivalent in WT and *Fgf21*^−*/*−^ mice indicating that the absence of FGF21 does not compromise this neuronal lineage (Figure 4B). These data demonstrate that FGF21 is required for ethanol to activate noradrenergic neurons in the LC.

### FGF21 acts directly on noradrenergic neurons to induce arousal from intoxication

**Figure 5.**
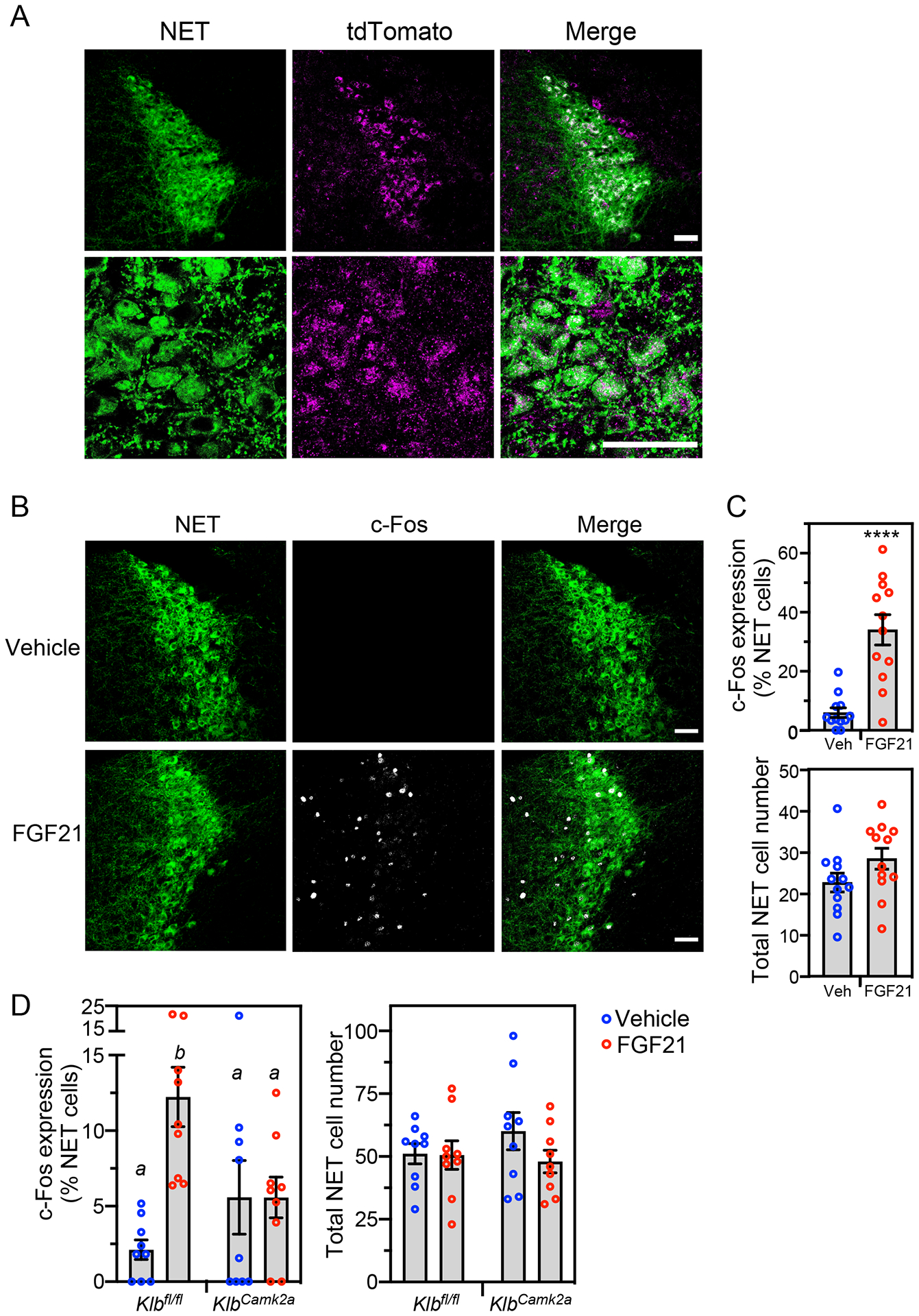
Pharmacologic FGF21 activates noradrenergic neurons in the locus coeruleus. (A) Confocal images of immunostaining performed in locus coeruleus (LC) sections prepared from transgenic mice expressing tdTomato fused to the C-terminus of KLB. Antibodies against tdTomato (magenta) and norepinephrine transporter (NET; green) were used. Scale bars represent 50 μM. (B and C) Immunostaining for c-Fos (white) and NET (green) in LC sections prepared from wild-type mice treated for 2 hours with vehicle or FGF21 (1 mg/kg, i.p.). Representative confocal images are shown in (B). Scale bars represent 50 μM. Quantification of c-Fos/NET co-expression (top panel) and total NET-positive cell number (lower panel) is shown in (C) (n = 4 sections/mouse, 3 mice/group). Data represent the mean ± SEM. ****, *P* < 0.0001 by Student’s t-test. (D) Immunostaining for c-Fos and NET in LC sections from groups of control (*Klb*^*fl/fl*^) and neuron-specific *Klb*^−/−^ (*Klb*^*Camk2a*^) mice treated for 2 hours with vehicle or FGF21 as in (B). Quantification of c-Fos/NET co-expression (left panel) and total NET-positive cell number (right panel) is shown (n = 3 sections/mouse, 3 mice/group). Data represent the mean ± SEM. Different lowercase letters indicate statistical significance (*P* < 0.05) by two-way ANOVA. See also Figure S1.

To determine whether FGF21 acts directly on noradrenergic neurons, we first examined whether FGF21’s obligate co-receptor, KLB, which is much less broadly expressed in brain than FGFR1c^24,25^, is present in the LC. Due to the lack of reliable KLB antibodies, we measured KLB expression using an established reporter mouse in which tdTomato was knocked into the endogenous *Klb* gene, resulting in a KLB-tdTomato fusion protein^26^. The fusion protein was detected by immunostaining in the LC, where it co-localized with NET in many but not all cells (Figure 5A). The fusion protein was also detected in regions adjacent to the LC where there was little or no NET staining (Figure 5A). Thus, KLB is expressed in noradrenergic neurons and other cell types in and around the LC. Due to the lack of antibodies selective for FGFR1c, which is the second constituent of the FGF21 receptor complex, we examined its expression at the mRNA level by in situ hybridization. *Fgfr1c* mRNA was broadly expressed throughout the LC region, where it colocalized with *Klb* mRNA in some noradrenergic neurons (Figure S1). FGFR1c may serve as a receptor for FGFs other than FGF21 in those cells in which *Klb* is not co-expressed.

Consistent with the *Fgfr1c*/*Klb* expression data, pharmacologic FGF21 administration induced c-Fos immunoreactivity in NET^+^ LC neurons of WT mice (Figure 5B and 5C). When this same pharmacologic experiment was performed in neuron-specific *Klb*^*Camk2a*^ mice, there was no induction of c-Fos by FGF21 (Figure 5D).

**Figure 6.**
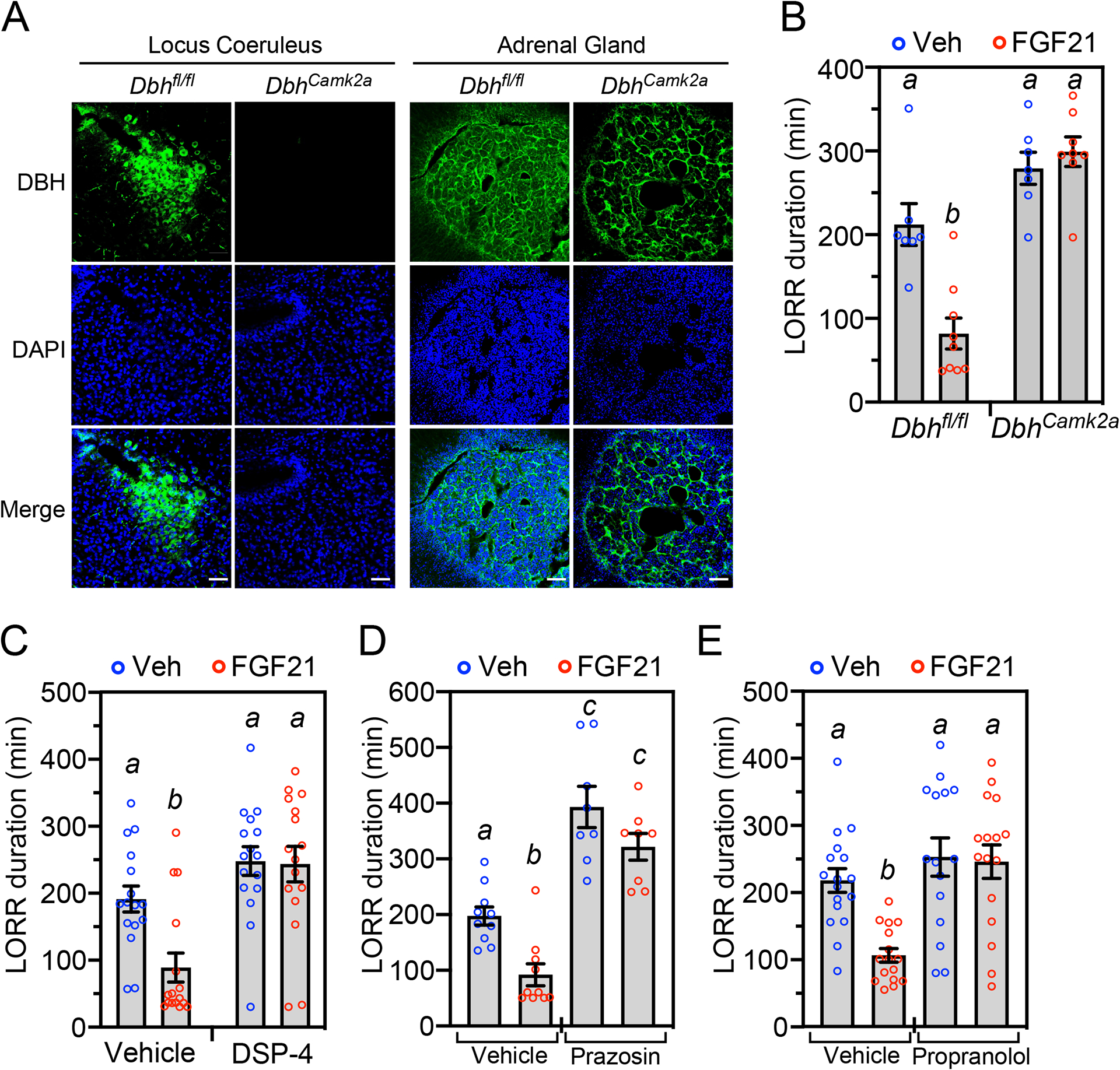
FGF21 exerts its anti-intoxicant activity through noradrenergic neurons. (A) Immunostaining for DBH in locus coeruleus and adrenal from control (*Dbh*^*fl/fl*^) and neuron-specific *Dbh*^−/−^ (*Dbh*^*Camk2a*^) mice. DAPI staining (blue) and merge of the two is shown below. Scale bars represents 50 μM. (B) *Dbh*^*fl/fl*^ and *Dbh*^*Camk2a*^ mice were administered ethanol (5 g/kg, oral gavage) followed 1 hour later by injection of vehicle or FGF21 (1 mg/kg, i.p.). Loss of righting reflex (LORR) duration was measured after FGF21 or vehicle administration (n = 7–9 mice/group). (C) Wild-type (WT) mice were injected with DSP-4 (50 mg/kg, i.p.) or vehicle. Two days later, the mice were administered ethanol (5 g/kg, oral gavage) followed 1 hour later by injection of FGF21 (1 mg/kg, i.p.) or vehicle (n = 16 mice/group). LORR duration was measured after FGF21 or vehicle administration. (D) WT mice were administered ethanol (5 g/kg, oral gavage) followed 1 hour later by injection of vehicle, FGF21 (1 mg/kg, i.p.), prazosin (0.8 mg/kg) or FGF21+prazosin (n = 8–10 mice/group). LORR duration was measured after vehicle or FGF21 administration. (E) The experiment was performed as in (B) except mice were injected with vehicle, FGF21 (1 mg/kg, i.p.), propranolol (10 mg/kg, i.p.) or FGF21+propranolol (n = 15–17 mice/group). LORR duration was measured after vehicle or FGF21 administration. All data represent the mean ± SEM. Different lowercase letters indicate statistical significance (*P* < 0.05) by two-way ANOVA. See also Figure S2.

To test whether noradrenergic neurons are required for FGF21’s anti-intoxicant activity, we employed complementary genetic knockout and pharmacologic inhibition approaches. To selectively eliminate NE production in neurons, we generated a floxed-*Dbh* mouse line (Figure S2) and crossed it with *Camk2a*-Cre mice. As expected, DBH expression was eliminated in the LC but not the adrenal medulla of the resulting *Dbh*^*Camk2a*^ mice (Figure 6A). Notably, these knockout mice were completely refractory to FGF21’s pharmacologic effect on righting reflex (Figure 6B). Likewise, pre-treatment of wild-type mice with DSP-4, a neurotoxin that readily crosses the blood–brain barrier and selectively and irreversibly inhibits NE signaling^27^, eliminated FGF21’s amethystic effect on righting reflex (Figure 6C). Upon its release from neurons, NE acts on α_1_- and β-adrenergic receptors in multiple subcortical regions to stimulate arousal^17^. FGF21’s anti-intoxicant effect was also blocked by the selective α_1_- and β-adrenergic receptor antagonists, prazosin and propranolol, respectively (Figures 6D and 6E). As previously reported, prazosin on its own also prolonged ethanol-induced loss of righting reflex (Figure 6D)^28^. Together, these genetic and pharmacologic data show that FGF21 stimulates arousal by activating noradrenergic neurons in the nervous system.

**Figure 7.**
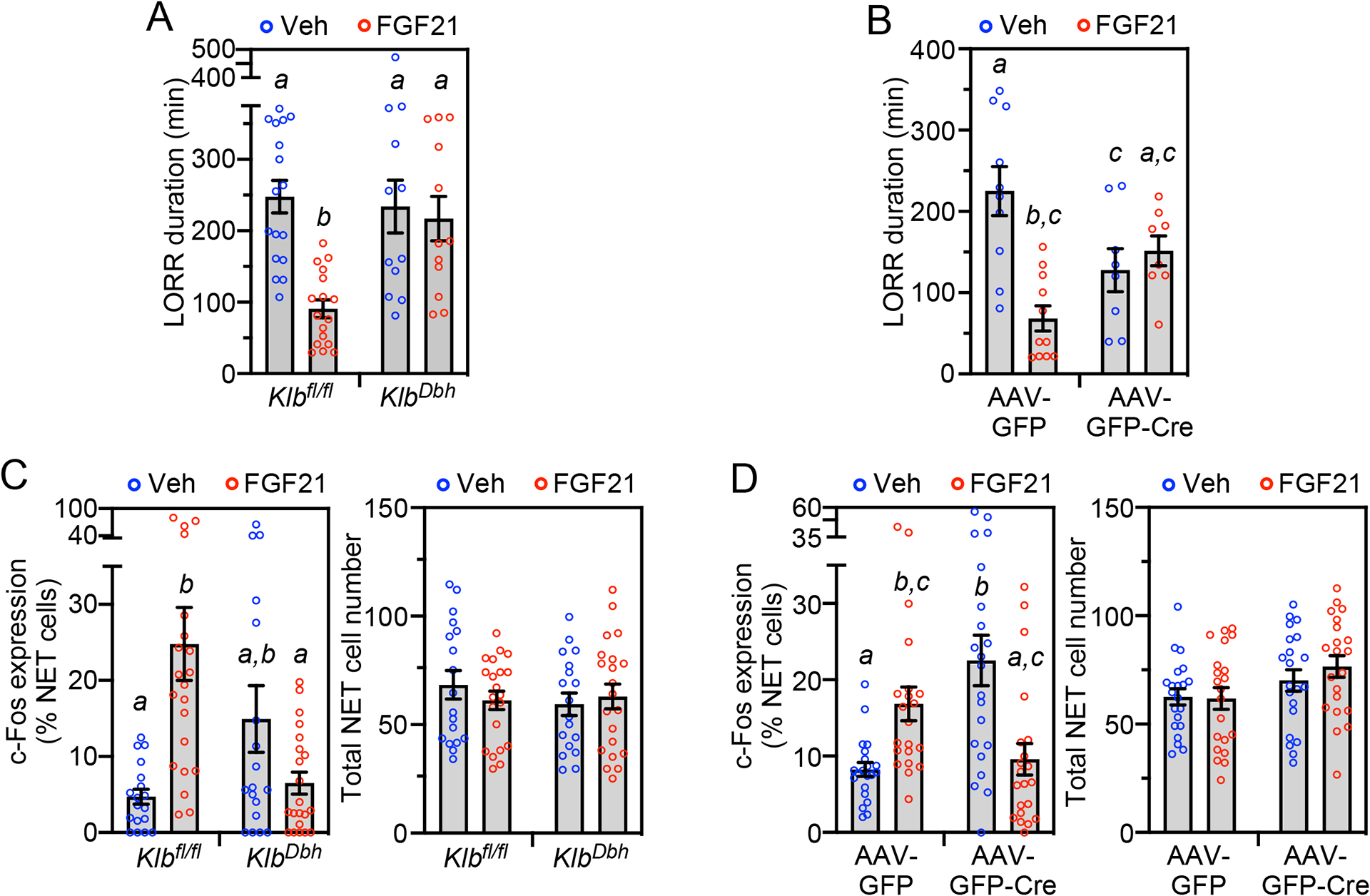
FGF21 acts directly on noradrenergic neurons in the locus coeruleus region. (A) Control (*Klb*^*fl/fl*^) and noradrenergic neuron-specific *Klb*^−/−^ (*Klb*^*Dbh*^) mice were administered ethanol (5 g/kg, oral gavage) followed 1 hour later by injection of vehicle or FGF21 (1 mg/kg, i.p.). LORR duration was measured after FGF21 or vehicle administration (n = 12–17 mice/group). (B) *Klb*^*fl/fl*^ mice bilaterally injected in the locus coeruleus (LC) region with adeno-associated viruses (AAV) expressing either control (GFP) or GFP-Cre were administered ethanol (5 g/kg, oral gavage) followed 1 hour later by injection of vehicle or FGF21 (1 mg/kg, i.p.). LORR duration was measured after FGF21 or vehicle administration (n = 8–11 mice/group). (C, D) Immunostaining for c-Fos and norepinephrine transporter (NET) was performed in LC sections from groups of control (*Klb*^*fl/fl*^) and *Klb*^*Dbh*^ mice (C) or *Klb*^*fl/fl*^ mice bilaterally injected in the LC region with AAV-GFP or AAV-GFP-Cre (D) treated for 2 hours with vehicle or FGF21 (1 mg/kg, i.p.). Quantification of c-Fos/NET co-expression (left panel) and total NET-positive cell number (right panel) is shown. For (C), n = 3 sections/mouse, 6–7 mice/group. For (D), n = 7 sections/mouse, 3 mice/group. All data represent the mean ± SEM. Different lowercase letters indicate statistical significance (p < 0.05) by two-way ANOVA. See also Figure S3.

We next performed *Klb* knockout studies to examine whether FGF21 acts directly on noradrenergic neurons in the LC region. *Klb*^*fl/fl*^ mice were crossed with *Dbh*-Cre mice to selectively disrupt FGF21 activity in noradrenergic neurons. In separate experiments, adenovirus-associated viruses (AAVs) expressing either Cre recombinase fused with green fluorescent protein (GFP) or GFP alone were bilaterally injected into the LC region. Immunostaining of representative brain sections with a GFP antibody showed successful targeting of the LC region in *Klb*^AAV-*Cre*^ mice (Figure S3A), and representative groups of both *Klb*^*Dbh*^ and *Klb*^AAV-*Cre*^ mice had significantly reduced *Klb* mRNA in the LC region but not in the suprachiasmatic nucleus or liver, where *Klb* is also expressed (Figure S3B). Consistent with the *Dbh* knockout and pharmacologic inhibitor studies above, FGF21’s effect on righting reflex was abolished in both *Klb*^*Dbh*^ and *Klb*^AAV-*Cre*^ mice (Figures 7A and 7B). Ethanol-induced hypothermia was unchanged in *Klb*^*Dbh*^ compared to control mice both in the presence and absence of FGF21 (Figure S3C), demonstrating that these righting reflex recovery data were not confounded by impaired thermoregulation. The ability of FGF21 to stimulate c-Fos expression in the LC was also abolished in both *Klb*^*Dbh*^ and *Klb*^AAV-*Cre*^ mice (Figures 7C and 7D). However, there were two unexpected results. First, *Klb*^AAV-*Cre*^ mice recovered their righting reflex significantly faster than control mice (Figure 7B) and had a corresponding increase in basal c-Fos expression in noradrenergic LC neurons (Figure 7D). This may reflect the noradrenergic nervous system’s robust ability to compensate for perturbations in its activity with changes at multiple levels, including norepinephrine release, post-synaptic receptor number and downstream signaling pathways^29^. Second, pharmacologic FGF21 decreased c-Fos expression in both *Klb*^*Dbh*^ and *Klb*^AAV-*Cre*^ mice (Figures 7D), indicating indirect effects of FGF21 on LC neurons when *Klb* expression is eliminated in either LC or all noradrenergic neurons. Accordingly, the FGF21 receptor complex is abundantly expressed in other brain regions such as the nucleus of the solitary tract that can impact LC activity^30^. While additional studies will be required to dissect the details of the underlying mechanisms, we conclude from these genetic knockout studies that FGF21 accelerates righting reflex recovery by acting directly on noradrenergic neurons, and that deletion of KLB in the LC region alters both the basal and FGF21-induced amethystic response.

## DISCUSSION

The hormone FGF21 is strongly induced in liver by both ethanol and its fermentation precursor, fructose^5–7,31–33^. In this report, we show that in addition to suppressing ethanol preference, stimulating water consumption and protecting against liver injury, FGF21 also protects against ethanol-induced loss of righting reflex and impairment of balance via direct effects on the noradrenergic nervous system. FGF21 does this without changing the rate at which ethanol is catabolized. Surprisingly, FGF21 does not counteract the loss of righting reflex caused by other sedatives, including ketamine, diazepam and pentobarbital. Taken together, this work reveals that FGF21 is an endogenous, ethanol-selective amethystic agent that complements the liver’s alcohol metabolizing enzymes in defending against ethanol toxicity and its potentially dangerous sequelae.

Previous studies in mice and rats showed that systemic ethanol administration acutely activates neurons in the LC^20–22^. We show that this stimulatory effect requires FGF21. Our results using liver-specific *Fgf21*^−*/*−^ mice and neuron-specific *Klb*^*Camk2a*^ mice support a model in which liver-derived FGF21 acts directly on the nervous system to counteract ethanol-induced intoxication. Using *Dbh*^*Camk2a*^ and *Klb*^*Dbh*^ knockout mice and the pharmacologic inhibitor DSP-4, we further show that FGF21 exerts its anti-intoxicant effect by acting directly on noradrenergic neurons, which regulate arousal and alertness. Noradrenergic neurons in the LC have been shown to be more active during periods of wake than sleep, and selective optogenetic stimulation of LC neurons elicits an immediate sleep-to-wake transition^34^. NE stimulates arousal in part through α_1_ and β-receptors in several brain regions, including the medial septal and medial preoptic areas^17^. Accordingly, we show that FGF21’s amethystic activity is abolished genetically by selectively disrupting *Klb* expression in the LC region and pharmacologically by administering α_1_ and β-adrenergic receptor antagonists.

Previous genetic and pharmacologic studies established that activation of NE neurons counteracts ethanol intoxication. Global DBH knockout mice exhibit the same prolonged righting reflex recovery time in response to binge ethanol that we observe in *Fgf21*^−*/*−^ mice, and restoration of central NE rescues this phenotype^23^. Likewise, drugs that suppress NE concentrations or downstream cAMP signaling also affect the degree of intoxication. For example, the tyrosine hydroxylase inhibitor, α-methyl-p-tyrosine, and the α-adrenergic receptor antagonist, phentolamine, increased ethanol-induced sleep time in mice^35–37^. α-Methyl-p-tyrosine also increased ethanol-induced impairment of psychomotor performance and reaction time in humans^38^. Conversely, intracerebroventricular administration of dibutyryl cAMP antagonized ethanol-induced sedation in rats in a dose-dependent manner^39^. Interestingly, impaired cAMP signaling increases the sensitivity of *Drosophila* to ethanol intoxication^40^, suggesting that the mechanism underlying anti-intoxicant activity may be evolutionarily conserved.

In addition to ethanol and fructose, FGF21 is also induced by starvation and low-protein diets^41–43^. We speculate that the FGF21-NE pathway may also heighten arousal and alertness in order to increase foraging during periods of nutritional deficiency. Consistent with this, we previously showed that FGF21 increases wheel-running activity in mice during the light phase, which is highly unusual behavior for these nocturnal animals^30^. In addition to arousal, the noradrenergic nervous system impacts myriad other neuronal processes including attention, memory, perception, and motivation^17,18^. Thus, the FGF21-NE pathway may modulate a variety of cognitive and affective functions to enhance survival under stressful conditions.

In summary, FGF21 serves as an endogenous hormonal signal from liver to noradrenergic neurons in the brain to defend against ethanol-induced intoxication. Post hoc pharmacologic administration of FGF21 also markedly accelerates arousal from ethanol’s detrimental effects on righting reflex and rotarod performance. These results reveal a mechanism for selectively targeting noradrenergic neurons that could prove useful for treating both the loss of consciousness and impaired mobility that occur during acute alcohol poisoning.

### Limitations of the Study

Our studies implicate noradrenergic neurons in the LC region of the brain as important targets for FGF21’s anti-intoxicant activity in mice. However, our AAV-Cre injection experiments do not rule out the possibility that non-noradrenergic *Klb*^+^ neurons in the LC or adjacent regions contribute to FGF21’s amethystic effects. Moreover, we are unable to explain at present why basal righting reflex recovery from ethanol intoxication is accelerated and pharmacologic FGF21 administration represses c-Fos expression in LC noradrenergic neurons in these *Klb*^AAV-*Cre*^ mice, although other experimental perturbations of the noradrenergic nervous system elicit strong compensatory responses^29^. It remains to be determined whether activation of the noradrenergic system contributes to other effects of FGF21, including those on metabolism and ethanol and sweet preference. Likewise, although both FGF21 and noradrenergic nervous system activity are induced by ethanol in humans^5–7,44^, additional studies will be required to determine whether FGF21’s anti-intoxicant activity translates to humans.

## RESOURCE AVAILABILITY

### Lead Contact

Further information and requests for resources and reagents should be directed to and will be fulfilled by the lead contact, Steven A. Kliewer (steven.kliewer@utsouthwestern.edu).

### Materials Availability

All reagents generated in this study are available from the lead contact with a completed Materials Transfer Agreement.

### Data and Code Availability

- All data points used to create the graphs can be found in Data S1.
- This paper does not report original code.
- Any additional information required to reanalyze the data reported in this paper is available from the lead contact upon request.

## EXPERIMENTAL MODEL DETAILS

### Mouse models

All use of mice and related procedures were approved by the University of Texas Southwestern Medical Center’s Institutional Animal Care and Use Committee. Mice were housed in a temperature-controlled environment (23 ± 1° C) with 12 hour light/dark cycles and fed standard rodent chow ad libitum. Mice were randomly assigned to experimental groups. Experiments were performed with male mice unless indicated otherwise. *Klb*^*Dbh*^ mice were generated by crossing *Klb*^*fl/fl*^ mice^30^ with *Dbh*-Cre mice (Jackson Laboratory, Stock No: 033951). *Fgf21*^−/− 45^, *Fgf21*^*Alb* 7^, and KLB-T mice^26^ were on a C57BL/6J background and *Klb*^*Camk2a* 30^ and *Klb*^*Dbh*^ mice were on mixed C57BL/6J;129/Sv backgrounds.

### Generation of *Dbh*^*fl/fl*^ mice

*Dbh*^*fl/fl*^ mice, in which Exon 3 of the *Dbh* gene is flanked by loxP sites, were generated using a CRISPR/Cas-9 gene editing strategy^46^. Briefly, two crRNAs (5’-ACTCACCATTGAACCTATGC-3’ for 5’ LoxP and 5’-ACCTGGGTCCCAGAGTTGCA-3’ for 3’ LoxP) and one tracrRNA were used. Two ssODNs, 5’-caggcagagagtgttatggtcttctcatttgctaagcggacagcgaggagcttcacttggtaggaggtcatgtg acatgattctcttcactcaccattgaacctgaattcgttgcgtgaATAACTTCGTATAATGTATGCTATACGAAGT TATatgctggctctgagcgggcaatcaactggttctgtctggctacagg-3’ and 5’-cactgagctacccccacgcttcccccgacaccacatcatcatggtaaacgggggtagagctctgctttccacctgggtcccagagtATA ACTTCGTATAATGTATGCTATACGAAGTTATgttgcgtgaggattctgcatggatcgaggtgctaccctggctccttagaagtagcacatatg-3’ were used as a transgene. We used 5’-gtgcttaacggtgaggacagg-3’ and 5’-ctgtatgcaggcctgaggtg-3’ primers for detecting 5’ LoxP insertion, 5’-cgacaatgagaccacgtactgg-3’ and 5’- ggaaatattcatctcaggggccc-3’ primers for detecting 3’ LoxP insertion, and 5’-ggcagagagtgttatggtc-3’ and 5’-gttctgttacctcctggctctg-3’ primers for sequence confirmation. DNA and RNA oligonucleotides were purchased from Integrated DNA Technologies, Inc. (Coraville, IA, USA). Two crRNAs, one tracrRNA, two ssODNs, and Cas9 protein were co-injected into fertilized oocytes from C57BL/6N female mice by the University of Texas Southwestern Transgenic Core. Founder mice were bred with C57BL/6J mice and progeny screened for the correct mutation by PCR and sequencing. After back-crossing founder mice with C57BL/6J mice for three generations, *Dbh*^*fl/+*^ mice were mated to generate homozygous *Dbh*^*fl/fl*^ mice.

## METHOD DETAILS

### AAV Injections

*Klb*^*fl/fl*^ mice were anesthetized using isoflurane anesthesia (3%−4% for induction; 1.5%−2% for maintenance) and positioned in a stereotaxic instrument (David Kopf Instruments) with a temperature controller to maintain body temperature. The skull was exposed, bregma was determined, and two small holes were drilled for bilateral injection into the LC (coordinates from lambda: ±0.9 mm mediolateral, −0.9 mm antero-posterior and −3.82 mm dorso-ventral^47^). Dorso-ventral coordinates are relative to pia. A volume of 300 nl (1 × 10^12^ genomic particles/μl) of AAV8-GFP or AAV8-GFP-Cre virus (UNC Vector Core) was injected bilaterally into the LC. The virus was infused at 50 nl/minute using a microinjection syringe pump and Micro2T controller system with a 34G Nanofil needle (World Precision Instrument, UMP3T-1). After each injection, the needle was maintained in position for 10 minutes to prevent backflow and then slowly removed over 5 minutes. The skin was closed using sutures. Mice were allowed to recover for two weeks before use in experiments.

### Loss of Righting Reflex (LORR) Studies

Time to LORR was defined as the time between ethanol administration to mice and LORR. Unless indicated otherwise, ethanol was administered by oral gavage in a volume of 0.02 ml/g to a final concentration of 5 g/kg. Once ataxic, mice were placed in a supine position in V-shaped plastic troughs and the time measured until they were able to right themselves three times within 30 seconds, which was defined as LORR duration. LORR studies were performed in 2–8-month-old mice. For studying FGF21’s pharmacologic effects on ethanol-induced LORR, mice were administered ethanol followed 1 hour later by injection of either FGF21 or vehicle. Unless indicated otherwise, FGF21 was i.p. injected in a volume of 0.01 ml/g to a final concentration of 1 mg/kg. For the sedatives study, ketamine (200 mg/kg), diazepam (30 mg/kg), pentobarbital (55 mg/kg) or ethanol (4.3 g/kg) were i.p. injected followed 30 minutes later by injection of FGF21 or vehicle. Diazepam and ketamine were diluted in 0.9% saline, and pentobarbital was dissolved in 0.9% saline containing 10% ethanol. Ketamine, diazepam and pentobarbital were injected in a volume of 0.01 ml/g, and ethanol was injected in a volume of 0.017 ml/g.

### DSP-4, Prazosin and Propranolol Studies

For the DSP-4 studies, 2-month-old C57BL/6J mice were i.p. injected with either DSP-4 (50 mg/kg in a volume of 0.01 ml/g) or vehicle. Two days later, mice were administered ethanol followed 1 hour later by injection of either FGF21 or vehicle. For the prazosin and propranolol studies, 2–3-month-old mice were administered ethanol followed 1 hour later by injection of FGF21 or vehicle in the presence or absence of prazosin (0.8 mg/kg in a volume of 0.01 ml/g) or propranolol (10 mg/kg in a volume of 0.01 ml/g). DSP-4, prazosin and propranolol were all dissolved in 0.9% saline just prior to use.

### Rotarod Studies

Three-to-six-month-old mice were trained for three consecutive days on a Rotamex-5 rotarod (Columbus Instruments) spinning at 5 rpm, with training complete when mice were able to stay on the rotarod without falling completely off for 60 seconds. On the fourth day, mice were i.p. injected with ethanol (2 g/kg in a volume of 0.014 ml/g) followed 30 minutes later by injection of either FGF21 or vehicle. Time on the rotarod spinning at 5 rpm until falling completely off, with a maximum of 60 seconds, was measured at regular intervals.

### Rectal Temperature Measurement

Rectal temperature was measured using a BAT-12 Microprobe digital thermometer with a RET-3 mouse rectal probe (Physitemp Instruments, Clifton, New Jersey). Mice (2–4-month-old) were gently restrained, and the lubricated probe was inserted 1.5 cm into the rectal cavity to determine body temperature.

### c-Fos Induction and Immunohistochemistry

For the c-Fos induction studies, 2–7-month-old mice were habituated for four days by either i.p. injection of 0.9% saline or oral gavage with water. On the fifth day, mice were i.p. injected with vehicle or FGF21 (2 hour treatment), or orally gavaged with water or ethanol as indicated in the figure legends. For all immunohistochemistry experiments, mice were anesthetized with isoflurane and transcardially perfused first with PBS followed by 10% neutral buffered formalin (NBF). Brains or adrenals were fixed for 24 hours in 10% NBF at 4°C and 50 μm slices were prepared using a Leica VT1000S vibratome. Slices were incubated for 1 hour in blocking buffer (1% bovine serum albumin, 5% normal goat serum, 0.3% Triton X-100 in PBS) at room temperature with shaking followed by incubation in primary antibodies, including antibodies against NET (Mab Technologies, 1:1000 dilution), cFos (Cell Signaling Technology, 1:1000 dilution), red fluorescent protein (Rockland, 1:500 dilution), GFP (Aves Labs, 1:2000 dilution) and DBH (MilliporeSigma, 1:1000 dilution) for 48 hours at 4°C. Free-floating slices were washed 3 times in PBS for 10 minutes followed by incubation for 1 hour at room temperature with Alexa Fluor-conjugated secondary antibodies, including goat anti-mouse, goat anti-chicken, goat anti-rabbit IgGs (Invitrogen, 1:500 dilution), and DAPI (Fisher Scientific, 1:5000 dilution) in blocking buffer. Slices were washed 3 times for 10 minutes in PBS and mounted with Aqua-Poly/Mount (Polysciences). Images were taken using a Zeiss LSM780 confocal microscope and images were processed using Fiji software^48^. c-Fos counts were performed blinded.

### Microdissection of Brain Regions and Quantitative PCR Analysis

Brains from 2–5-month-old mice were extracted from the skull and kept under dry ice vapor for all dissections. Coronal sections (0.5 mm thickness) were cut using a brain-slicing matrix (Braintree Scientific). SCN and LC regions were identified by gross anatomical landmarks^47^ and dissected using a 16G tissue punch. Tissue was homogenized by passage through a 26.5G syringe in RNA-STAT60. Total RNA was isolated from tissue using RNA-STAT60 reagent, and RNA was reverse-transcribed into cDNA (Invitrogen). Gene expression was measured with an Applied Biosystems 7900HT Sequence Detection System using the ddCT assay and normalized to cyclophilin^49^.

### In Situ Hybridization Analysis

C57BL/6J, 5-month-old mice were anesthetized with isoflurane and transcardially perfused first with PBS followed by 10% NBF. Brains were fixed for 24 hours at 4°C in 10% NBF and then switched to 30% sucrose for 24 hours. Brain slices (25 μm) were cut using a freezing microtome (Leica), collected in PBS and treated with hydrogen peroxide for 10 minutes. After a PBS rinse, slices were mounted and desiccated overnight at room temperature. In situ hybridization was performed using RNAScope multiplex fluorescence kits (cat# 323110) and *Klb* (cat# 415221 Mm-Klb) and *Fgfr1c* (cat# 454941-C2 Mm-Fgfr1-O1-C2) probes purchased from Advanced Cell Diagnostics. Hybridized slides were incubated with amplification reagents and Opal 570 and 690 dyes (Akoya Biosciences, 1:1500 dilution) followed by sequential incubation with tyrosine hydroxylase (Aves Labs, 1:1000 dilution), biotinylated anti-chicken secondary (Jackson ImmunoResearch, 1:1000 dilution) and streptavidin AlexaFluor488 (Invitrogen, 1:1000 dilution) antibodies. Slides were rinsed, dehydrated, cleared, and mounted with EcoMount (BioCare Medical). Images were taken using a Zeiss LSM880 confocal microscope.

### FGF21 and Ethanol Measurements

For measuring FGF21 and ethanol concentrations in murine plasma, blood from 4–6-month-old mice was centrifuged at 3,000 rpm for 15 minutes immediately after collection and plasma was stored at −80°C until analysis. Plasma FGF21 concentrations were measured using an FGF21 mouse/rat ELISA kit (BioVendor) according to the manufacturer’s instructions. Plasma ethanol concentrations were measured using an EnzyChrom ethanol assay kit (BioAssay Systems) according to the manufacturer’s instructions. For measuring brain ethanol concentrations, brains were removed, frozen immediately in liquid nitrogen and stored at −80°C. Frozen whole brains were homogenized in 0.1N HCl and centrifuged at 13,000 rpm for 30 minutes at 4°C. Ethanol concentrations in the supernatants were measured using an EnzyChrom ethanol assay kit.

## QUANTIFICATION AND STATISTICAL ANALYSIS

All data are expressed as the mean ± SEM. For Figures 1–3, 6B–E, 7A and B, and S3B and C, n is individual mice. In Figures 4B, 5C and D, and 7C and D, n is brain sections. Statistical analyses were performed using GraphPad Prism Software Version 9.0. Unpaired two-tailed Student’s t tests were used for two group analyses. Multiple groups were tested by one-way or two-way ANOVAs with Tukey’s multiple comparison test. For the rotarod analyses, a linear mixed effect model was fitted using the R package lme4 and group effect *P* values derived using a likelihood ratio test. In all analyses, a *P* value < 0.05 was considered significant. Data S1 contains the unprocessed data underlying the display items in the manuscript, related to Figures 1–7 and S3.

## Supplementary Material

1

2

3

## Figures and Tables

**Table:** Key resources table

REAGENT or RESOURCE	SOURCE	IDENTIFIER
Antibodies		
Rabbit monoclonal anti-cFos	Cell Signaling Technology	Cat# 2250S; RRID: AB_2247211
Mouse monoclonal anti-NET	Mab Technology	Cat# NET05-2; RRID: AB_2571639
Rabbit polyclonal anti-RFP	Rockland	Cat# 600-401-379; RRID: AB_2209751
Chicken polyclonal anti-GFP	Abcam	Cat# 13970; RRID: AB_300798
Rabbit polyclonal anti-DBH	Immunostar	Cat# 22806; RRID: AB_572229
Chicken polyclonal anti-TH	Aves labs	Cat# TYH; RRID: AB_10013440
Goat Anti-rabbit Alexa Fluor 594	Thermo Fisher Scientific	Cat# A11072; RRID: AB_2534116
Goat Anti-mouse Alexa Fluor 633	Thermo Fisher Scientific	Cat# A21052; RRID: AB_2535719
Goat Anti-chicken Alexa Fluor 594	Thermo Fisher Scientific	Cat# A11042; RRID: AB_2534099
Biotin-SP AffiniPure donkey anti-chicken secondary IgY	Jackson Immunoresearch	Cat# 703-065-155; RRID: AB_2313596
Streptavidin, Alexa Fluor 488 conjugate	Thermo Fisher Scientific	Cat# S11223
Bacterial and virus strains		
AAV8-hSyn-EGFP	UNC Vector Core	N/A
AAV8-hSyn-GFP-Cre	UNC Vector Core	N/A
		
		
		
Biological samples		
		
		
		
		
		
Chemicals, peptides, and recombinant proteins		
Recombinant human FGF21	Novo Nordisk	N/A
Ethanol (200 proof ethyl alcohol)	Pharmco-Aaper	C16A0720P
Prazosin hydrochloride	Sigma-Aldrich	Cat# P7791-50MG
Propranolol	TOCRIS Bioscience	Cat# 0624
N-(2-Chloroethyl)-N-ethyl-2-bromobenzylamine hydrochloride (DSP-4)	Sigma-Aldrich	Cat# C8417-100MG
Hydrochloric acid	EMD	Cat# HX0603-3
Ketamine (Ketaset)	Zoetis	Cat# 10004027
Diazepam	Hospira	Cat# 00409321312
Pentobarbital (Euthasol solution)	USP	Cat# VINV-CIII-0001
10% Buffered Formalin Phosphate	Thermo Fisher Scientific	Cat# SF100-4
Critical commercial assays		
Mouse FGF21 ELISA	Biovendor	Cat# RD291108200R
EnzyChrom Ethanol Assay Kit	BioAssay Systems	Cat# ECET-100
RNAscope multiplex fluorescence kits	Advanced Cell Diagnostics	Cat# 323110
RNAscope probe – Mm-Klb	Advanced Cell Diagnostics	Cat# 415221
RNAscope probe – Mm-Fgfr1-O1-C2	Advanced Cell Diagnostics	Cat# 454941
Opal 570 reagent	Akoya Biosciences	Cat# FP1488001KT
Opal 690 reagent	Akoya Biosciences	Cat# FP1497001KT
EcoMount	Biocare Medical	EM897L
Alt-R S.p. Cas9 Nuclease V3, 100ug	IDT	Cat# 1081058
Deposited data		
		
		
		
		
		
Experimental models: Cell lines		
		
		
		
		
		
Experimental models: Organisms/strains		
Mouse: C57BL/6J wild-type	The Jackson Laboratory	JAX: 000664
Mouse: DBH^Cre^ KI	The Jackson Laboratory	JAX: 033951
Mouse: FGF21 KO	Potthoff et al.^45^	N/A
Mouse: Klb^fl/fl^	Bookout et al.^30^	N/A
Mouse: Klb^Camk2a^	Bookout et al.^30^	N/A
Mouse: FGF21^Alb^	Song et al.^7^	N/A
Mouse: KLB-T	Coate et al.^26^	N/A
Mouse: DBH^fl/fl^	This paper	N/A
Mouse: DBH^Camk2a^	This paper	N/A
Oligonucleotides		
DBH3_5’_crRNA: ACTCACCATTGAACCTATGC	IDT	N/A
DBH3_3’_crRNA: ACCTGGGTCCCAGAGTTGCA	IDT	N/A
DBH3_5’_ssODN: caggcagagagtgttatggtcttctcatttgctaagcggacagcgaggagcttcacttggtaggaggtcatgtgacatgattctcttcactcaccattgaacctgaattcgttgcgtgaATAACTTCGTATAATGTATGCTATACGAAGTTATatgctggctctgagcgggcaatcaactggttctgtctggctacagg	IDT	N/A
DBH3_3’_ssODN: cactgagctacccccacgcttcccccgacaccacatcatcatggtaaacgggggtagagctctgctttccacctgggtcccagagtATAACTTCGTATAATGTATGCTATACGAAGTTATgttgcgtgaggattctgcatggatcgaggtgctaccctggctccttagaagtagcacatatg	IDT	N/A
DBH3_5’_LoxP PCR (Forward) gtgcttaacggtgaggacagg (Reverse) ctgtatgcaggcctgaggtg	IDT	N/A
DBH3_3’_LoxP PCR (Forward) cgacaatgagaccacgtactgg (Reverse) ggaaatattcatctcaggggccc	IDT	N/A
DBH3_5’ Sequence: ggcagagagtgttatggtc	IDT	N/A
DBH3_3’ Sequence: gttctgttacctcctggctctg	IDT	N/A
Alt-R CRISPR-Cas9 tracrRNA, 20nmol	IDT	Cat# 1072533
Recombinant DNA		
		
		
		
		
		
Software and algorithms		
GraphPad Prism 9	GraphPad	https://www.graphpad.com
Fiji	Schindelin et al.^48^	https://imagei.net/software/fiii/; RRID: SCR_002285
CCTop-CRISPR/Cas9 target online predictor		https://cctop.cos.uni-heidelberg.de:8043/
		
		
Other		
QPCR	Applied Biosystems	7900HT Sequence Detection System
Model 942 Small Animal Stereotaxic Instrument with Digital Display Console	David Kopf Instruments	https://kopfinstruments.com/product/model-942-small-animal-stereotaxic-instrument-with-digital-display-console/
Microinjection syringe pump and Micro2T controller system (UMP3T-1)	World Precision Instrument	https://www.wpiinc.com/var-8091-microinjection-syringe-pump-with-smartouch-controller?gclid=Cj0KCQiAz9ieBhCIARIsACB0oGIegIjgHutnBAF0MgC-PPc273tfDvE7LntYr2wnzWTsFuliDH1BYUMaAoEREALw_wcB
Rota Rod Rotamex-5	Columbus instruments	https://www.colinst.com/products/rota-rod-rotamex
Leica VT1000S vibrating blade microtome	Leica Biosystems	https://www.leicabiosystems.com/us/research/vibratomes/leica-vt1000-s/
Zeiss LSM780 confocal microscope	Zeiss	https://www.zeiss.com/microscopy/en/resources/insights-hub/raw-materials/ancient-fidget-spinner.html
Zeiss LSM880 confocal microscope	Zeiss	https://www.zeiss.com/microscopy/en/c/ind/22/zeiss-microscopy-image-contest-2022.html
Stainless Steel Brain Matrix	Braintree Scientific	https://www.braintreesci.com/neuroscience-physiology/brain-matrice-tissue-punches/stainless-steel-brain-matrice/
BAT-12 microprobe digital thermometer with a RET-3 mouse rectal probe	Physitemp Instruments	https://physitemp.com/electrotherms_p102
Standard rodent chow diet (Teklad Global 16% Protein Rodent Diet)	Teklad	https://www.envigo.com/rodent-natural-ingredient-2016-diets

## Highlights

- Hormone FGF21 counteracts alcohol-induced loss of consciousness and coordination
- Pharmacologic FGF21 accelerates recovery from alcohol-induced intoxication
- FGF21 exerts its sobering effect by activating the noradrenergic nervous system
