# Supplementary material for: FGF21 Counteracts Alcohol Intoxication by Activating the Noradrenergic Nervous System
Source: Cell Metab. Author manuscript; Available in PMC 2023 Mar 13. (PMC10009780; doi:10.1016/j.cmet.2023.02.005)
Supplement: 2 [file NIHMS1875761-supplement-2.docx]

***TABLE FOR AUTHOR TO COMPLETE***

*Please upload the completed table as a separate document.* ***Please do not add subheadings to the key resources table.*** *If you wish to make an entry that does not fall into one of the subheadings below, please contact your handling editor.* ***Any subheadings not relevant to your study can be skipped.*** *(****NOTE:*** *For authors publishing in Cell Genomics, Cell Reports Medicine, Current Biology, and Med, please note that references within the KRT should be in numbered style rather than Harvard.)*

**Key resources table**

| REAGENT or RESOURCE | SOURCE | IDENTIFIER |
| --- | --- | --- |
| Antibodies | | |
| Rabbit monoclonal anti-cFos | Cell Signaling Technology | Cat# 2250S; RRID: AB_2247211 |
| Mouse monoclonal anti-NET | Mab Technology | Cat# NET05-2; RRID: AB_2571639 |
| Rabbit polyclonal anti-RFP | Rockland | Cat# 600-401-379; RRID: AB_2209751 |
| Chicken polyclonal anti-GFP | Abcam | Cat# 13970; RRID: AB_300798 |
| Rabbit polyclonal anti-DBH | Immunostar | Cat# 22806; RRID: AB_572229 |
| Chicken polyclonal anti-TH | Aves labs | Cat# TYH; RRID: AB_10013440 |
| Goat Anti-rabbit Alexa Fluor 594 | Thermo Fisher Scientific | Cat# A11072; RRID: AB_2534116 |
| Goat Anti-mouse Alexa Fluor 633 | Thermo Fisher Scientific | Cat# A21052; RRID: AB_2535719 |
| Goat Anti-chicken Alexa Fluor 594 | Thermo Fisher Scientific | Cat# A11042; RRID: AB_2534099 |
| Biotin-SP AffiniPure donkey anti-chicken secondary IgY | Jackson Immunoresearch | Cat# 703-065-155; RRID: AB_2313596 |
| Streptavidin, Alexa Fluor 488 conjugate | Thermo Fisher Scientific | Cat# S11223 |
| Bacterial and virus strains | | |
| AAV8-hSyn-EGFP | UNC Vector Core | N/A |
| AAV8-hSyn-GFP-Cre | UNC Vector Core | N/A |
|  |  |  |
|  |  |  |
|  |  |  |
| Biological samples |  |  |
|  |  |  |
|  |  |  |
|  |  |  |
|  |  |  |
|  |  |  |
| Chemicals, peptides, and recombinant proteins | | |
| Recombinant human FGF21 | Novo Nordisk | N/A |
| Ethanol (200 proof ethyl alcohol) | Pharmco-Aaper | C16A0720P |
| Prazosin hydrochloride | Sigma-Aldrich | Cat# P7791-50MG |
| Propranolol | TOCRIS Bioscience | Cat# 0624 |
| N-(2-Chloroethyl)-N-ethyl-2-bromobenzylamine hydrochloride (DSP-4) | Sigma-Aldrich | Cat# C8417-100MG |
| Hydrochloric acid | EMD | Cat# HX0603-3 |
| Ketamine (Ketaset) | Zoetis | Cat# 10004027 |
| Diazepam | Hospira | Cat# 00409321312 |
| Pentobarbital (Euthasol solution) | USP | Cat# VINV-CIII-0001 |
| 10% Buffered Formalin Phosphate | Thermo Fisher Scientific | Cat# SF100-4 |
| Critical commercial assays | | |
| Mouse FGF21 ELISA | Biovendor | Cat# RD291108200R |
| EnzyChrom Ethanol Assay Kit | BioAssay Systems | Cat# ECET-100 |
| RNAscope multiplex fluorescence kits | Advanced Cell Diagnostics | Cat# 323110 |
| RNAscope probe – Mm-Klb | Advanced Cell Diagnostics | Cat# 415221 |
| RNAscope probe – Mm-Fgfr1-O1-C2 | Advanced Cell Diagnostics | Cat# 454941 |
| Opal 570 reagent | Akoya Biosciences | Cat# FP1488001KT |
| Opal 690 reagent | Akoya Biosciences | Cat# FP1497001KT |
| EcoMount | Biocare Medical | EM897L |
| Alt-R S.p. Cas9 Nuclease V3, 100ug | IDT | Cat# 1081058 |
| Deposited data | | |
|  |  |  |
|  |  |  |
|  |  |  |
|  |  |  |
|  |  |  |
| Experimental models: Cell lines | | |
|  |  |  |
|  |  |  |
|  |  |  |
|  |  |  |
|  |  |  |
| Experimental models: Organisms/strains | | |
| Mouse: C57BL/6J wild-type | The Jackson Laboratory | JAX: 000664 |
| Mouse: DBH^Cre^ KI | The Jackson Laboratory | JAX: 033951 |
| Mouse: FGF21 KO | Potthoff et al.^45^ | N/A |
| Mouse: Klb^fl/fl^ | Bookout et al.^30^ | N/A |
| Mouse: Klb^Camk2a^ | Bookout et al.^30^ | N/A |
| Mouse: FGF21^Alb^ | Song et al.^7^ | N/A |
| Mouse: KLB-T | Coate et al.^26^ | N/A |
| Mouse: DBH^fl/fl^ | This paper | N/A |
| Mouse: DBH^Camk2a^ | This paper | N/A |
| Oligonucleotides | | |
| DBH3_5’_crRNA: ACTCACCATTGAACCTATGC | IDT | N/A |
| DBH3_3’_crRNA: ACCTGGGTCCCAGAGTTGCA | IDT | N/A |
| DBH3_5’_ssODN: caggcagagagtgttatggtcttctcatttgctaagcggacagcgaggagcttcacttggtaggaggtcatgtgacatgattctcttcactcaccattgaacctgaattcgttgcgtgaATAACTTCGTATAATGTATGCTATACGAAGTTATatgctggctctgagcgggcaatcaactggttctgtctggctacagg | IDT | N/A |
| DBH3_3’_ssODN: cactgagctacccccacgcttcccccgacaccacatcatcatggtaaacgggggtagagctctgctttccacctgggtcccagagtATAACTTCGTATAATGTATGCTATACGAAGTTATgttgcgtgaggattctgcatggatcgaggtgctaccctggctccttagaagtagcacatatg | IDT | N/A |
| DBH3_5’_LoxP PCR  (Forward) gtgcttaacggtgaggacagg  (Reverse) ctgtatgcaggcctgaggtg | IDT | N/A |
| DBH3_3’_LoxP PCR  (Forward) cgacaatgagaccacgtactgg  (Reverse) ggaaatattcatctcaggggccc | IDT | N/A |
| DBH3_5’ Sequence: ggcagagagtgttatggtc | IDT | N/A |
| DBH3_3’ Sequence: gttctgttacctcctggctctg | IDT | N/A |
| Alt-R CRISPR-Cas9 tracrRNA, 20nmol | IDT | Cat# 1072533 |
| Recombinant DNA | | |
|  |  |  |
|  |  |  |
|  |  |  |
|  |  |  |
|  |  |  |
| Software and algorithms | | |
| GraphPad Prism 9 | GraphPad | https://www.graphpad.com |
| Fiji | Schindelin et al.^48^ | <https://imagej.net/software/fiji/>; RRID: SCR_002285 |
| CCTop-CRISPR/Cas9 target online predictor |  | https://cctop.cos.uni-heidelberg.de:8043/ |
|  |  |  |
|  |  |  |
| Other | | |
| QPCR | Applied Biosystems | 7900HT Sequence Detection System |
| Model 942 Small Animal Stereotaxic Instrument with Digital Display Console | David Kopf Instruments | https://kopfinstruments.com/product/model-942-small-animal-stereotaxic-instrument-with-digital-display-console/ |
| Microinjection syringe pump and Micro2T controller system (UMP3T-1) | World Precision Instrument | https://www.wpiinc.com/var-8091-microinjection-syringe-pump-with-smartouch-controller?gclid=Cj0KCQiAz9ieBhCIARIsACB0oGIegIjgHutnBAF0MgC-PPc273tfDvE7LntYr2wnzWTsFuliDH1BYUMaAoEREALw_wcB |
| Rota Rod Rotamex-5 | Columbus instruments | https://www.colinst.com/products/rota-rod-rotamex |
| Leica VT1000S vibrating blade microtome | Leica Biosystems | https://www.leicabiosystems.com/us/research/vibratomes/leica-vt1000-s/ |
| Zeiss LSM780 confocal microscope | Zeiss | https://www.zeiss.com/microscopy/en/resources/insights-hub/raw-materials/ancient-fidget-spinner.html |
| Zeiss LSM880 confocal microscope | Zeiss | https://www.zeiss.com/microscopy/en/c/ind/22/zeiss-microscopy-image-contest-2022.html |
| Stainless Steel Brain Matrix | Braintree Scientific | https://www.braintreesci.com/neuroscience-physiology/brain-matrice-tissue-punches/stainless-steel-brain-matrice/ |
| BAT-12 microprobe digital thermometer with a RET-3 mouse rectal probe | Physitemp Instruments | https://physitemp.com/electrotherms_p102 |
| Standard rodent chow diet (Teklad Global 16% Protein Rodent Diet) | Teklad | https://www.envigo.com/rodent-natural-ingredient-2016-diets |
