# Supplementary material for: FGF21 Counteracts Alcohol Intoxication by Activating the Noradrenergic Nervous System
Source: Cell Metab. Author manuscript; Available in PMC 2023 Mar 13. (PMC10009780; doi:10.1016/j.cmet.2023.02.005)
Supplement: 3 [file NIHMS1875761-supplement-3.pdf]

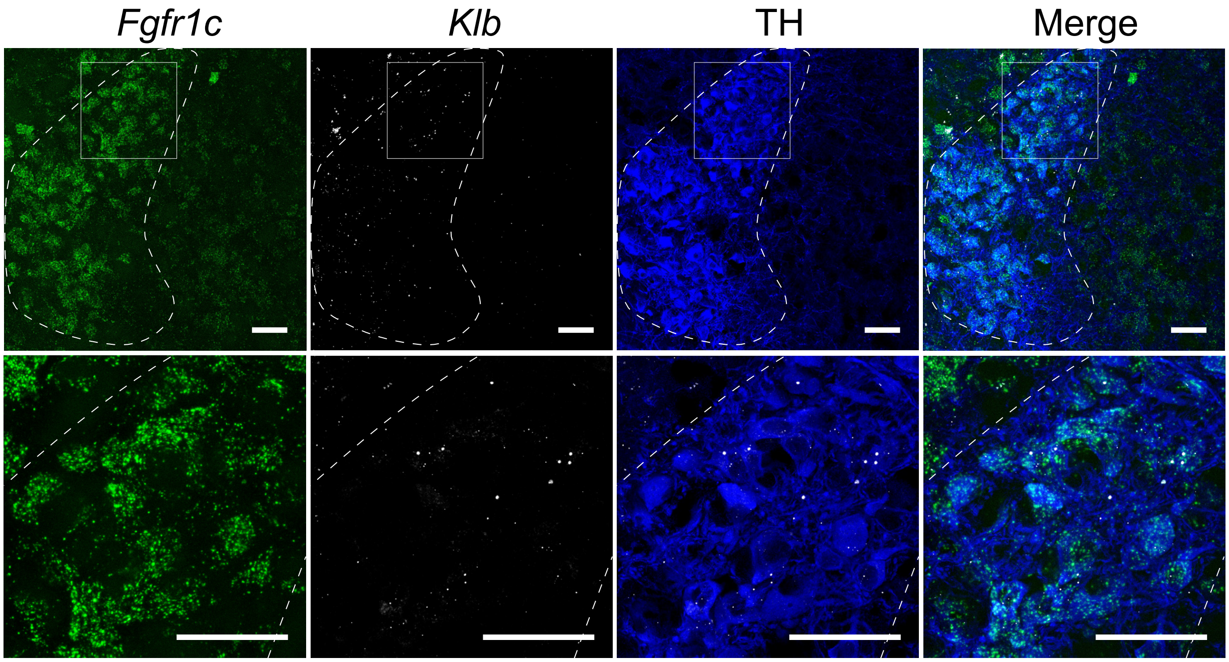

Figure S1, related to Figure 5. *Fgfr1c* and *Klb* are co-expressed in the locus coeruleus. In situ hybridization of *Fgfr1c* mRNA (green) and *Klb* mRNA (white), immunostaining for tyrosine hydroxylase (TH; blue), and the merge of the three. Two different magnifications are shown, with the boxed area in the top panels expanded in the bottom panels. Dotted lines outline the locus coeruleus. Scale bar represents 50 μM.

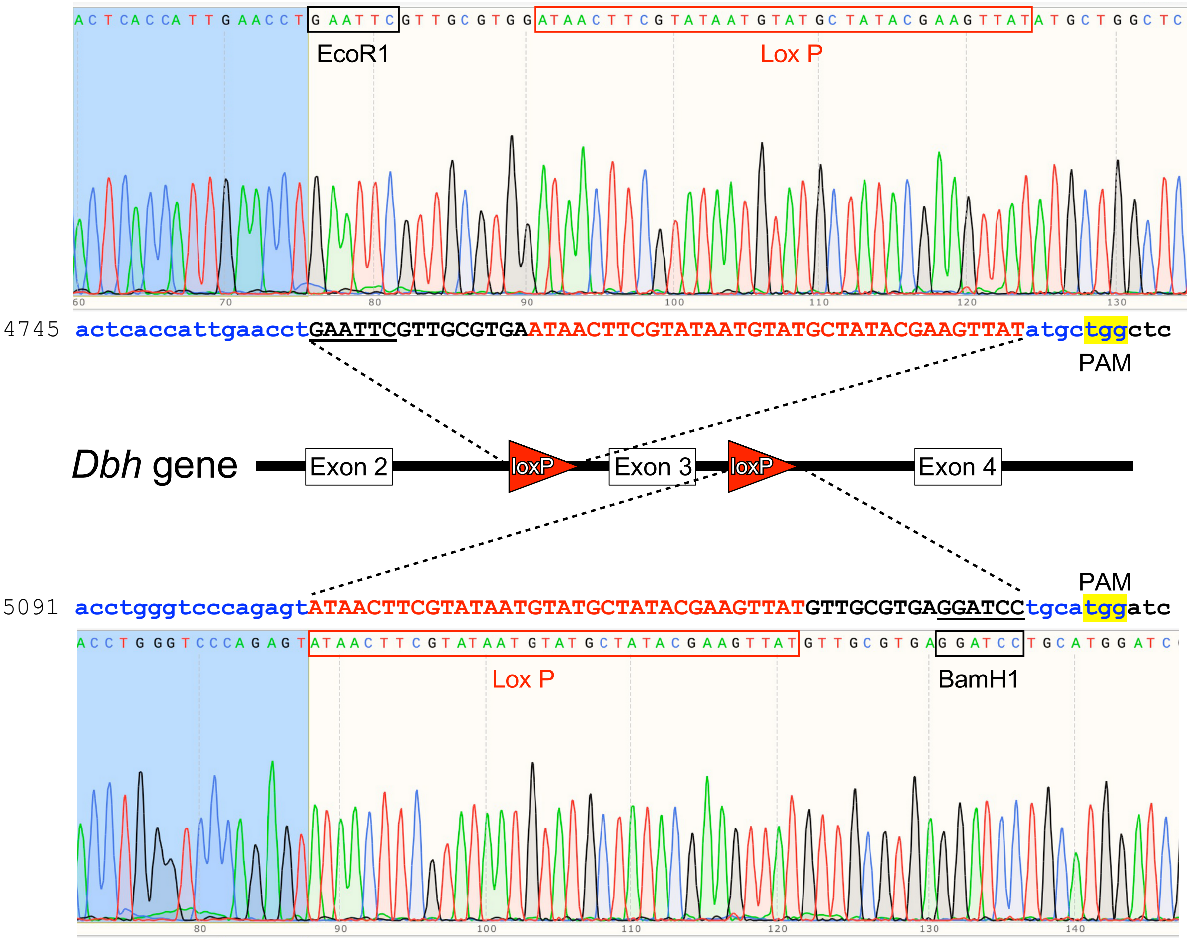

Figure S2, related to Figure 6. Strategy for generating the *Dbh*$^{fl/fl}$ mouse.

Schematic diagram shows the 5′ and 3′ *loxP* insertion sites flanking Exon 3 of the mouse *Dbh* gene and the inserted sequences. LoxP, Protospacer Adjacent Motifs (PAM) and restriction enzyme sites are indicated, with the genomic DNA targeted for recombination highlighted in blue.

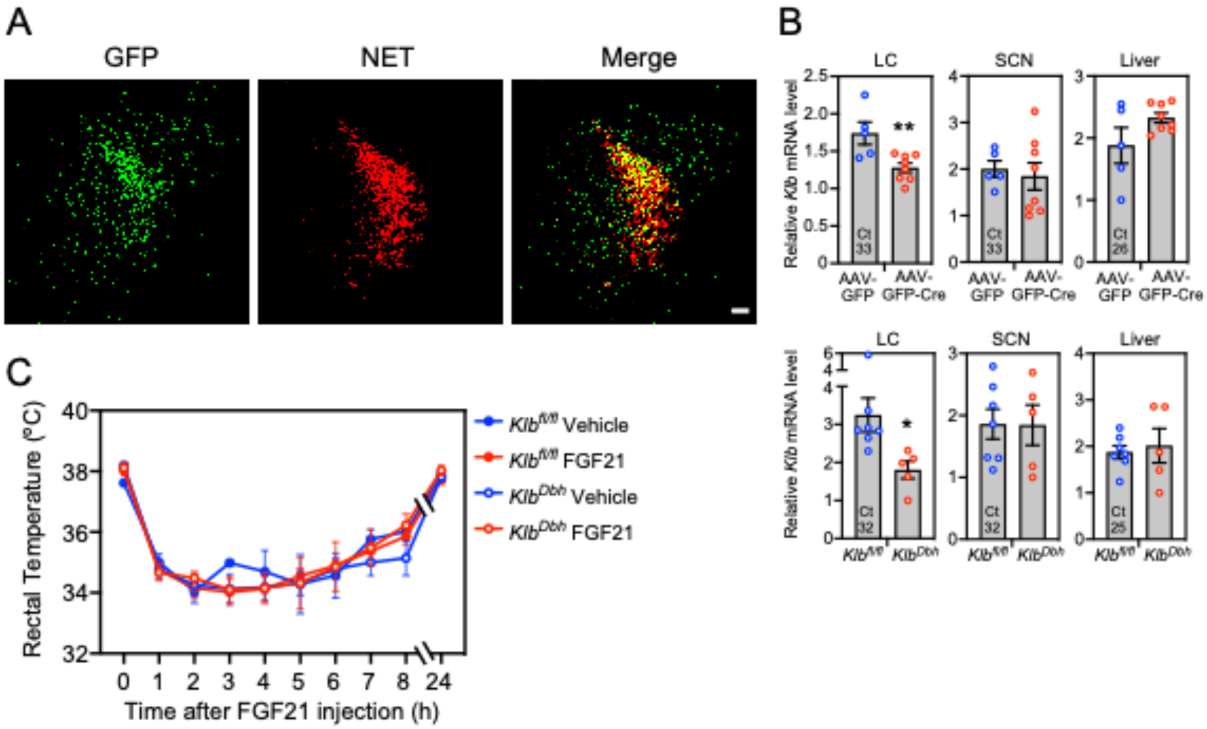

Figure S3, related to Figure 7. **Characterization of *Klb*^{Dbh} and *Klb*$^{AAV\text{-}Cre}$ mice.**

(A) Representative confocal image of immunostaining for GFP-Cre (green) and the norepinephrine transporter (NET; red) in a mouse stereotaxically injected in the locus coeruleus region with AAV-GFP-Cre. Scale bar represents 50 µM.

(B) *Klb* mRNA concentrations measured by qPCR in representative groups of *Klb*^{Dbh} and *Klb*$^{\text{AAV-}Cre}$ or control mice in the locus coeruleus (LC), suprachiasmatic nucleus (SCN) and liver. Ct values are shown (n = 5-8 mice/group). Data represent the mean ± SEM. *, $P < 0.05$, **, $P < 0.01$ compared to control mice by Student's t-test.

(C) Control (*Klb*$^{fl/fl}$) and noradrenergic neuron-specific *Klb*$^{-/-}$ (*Klb*^{Dbh}) mice were administered ethanol (5 g/kg, oral gavage) followed 1 hour later by injection of vehicle or FGF21 (1 mg/kg, i.p.). Core body temperature was measured by rectal thermometer at the indicated times (n = 6-7 mice/group).
